# Neurovascular Unit-Derived Extracellular Vesicles as Regulators of Post-Stroke Pathology and Neurorestoration

**DOI:** 10.3390/biom16030365

**Published:** 2026-02-28

**Authors:** Brianna Powell, Michael Chopp, Zhenggang Zhang, Xianshuang Liu

**Affiliations:** 1 Department of Neurology, Henry Ford Health, Detroit, MI 48202, USA; bpowell3@hfhs.org (B.P.); mchopp1@hfhs.org (M.C.); zzhang1@hfhs.org (Z.Z.); 2 Department of Physics, Oakland University, Rochester, MI 48309, USA

**Keywords:** neurovascular unit, extracellular vesicles, ischemic stroke, neurorestoration, blood–brain barrier, angiogenesis, neuroinflammation, white matter repair

## Abstract

Ischemic stroke is a leading cause of disability worldwide, marked by profound disruption of the neurovascular unit (NVU), a dynamic grouping of neurons, astrocytes, cerebral endothelial cells (CECs), microglia, pericytes, and oligodendrocytes. While acute stroke interventions such as tissue plasminogen activator and endovascular thrombectomy address reperfusion, they fail to engage the prolonged and cell-specific processes critical for recovery. Extracellular vesicles (EVs), membrane-bound carriers of proteins, lipids, and nucleic acids, have emerged as key modulators of intercellular communication within the NVU. This review synthesizes current evidence on NVU-derived EVs as both regulators and effectors of post-stroke pathology and repair. We highlight the phase-specific roles of EVs in modulating blood–brain barrier (BBB) integrity, thrombosis, angiogenesis, neurogenesis, oligodendrogenesis, synaptic plasticity, and neuroinflammation. This review places special emphasis on how EV cargo reflects the state of their parent cells and how EV-mediated crosstalk orchestrates coordinated neurorestorative responses. We further discuss the dual nature of EVs, their therapeutic potential for stroke, and the methodological challenges impeding clinical translation, including isolation standardization, cell-specific targeting, and regulatory barriers. Thus, adherence to minimal information for studies of extracellular vesicles (MISEV) guidelines is essential to ensure rigor, reproducibility, and transparency. When combined with temporal and cellular specificity, NVU-derived EVs may represent a biomimetic platform for promoting durable recovery in stroke patients.

## 1. Introduction

**Figure 1:**
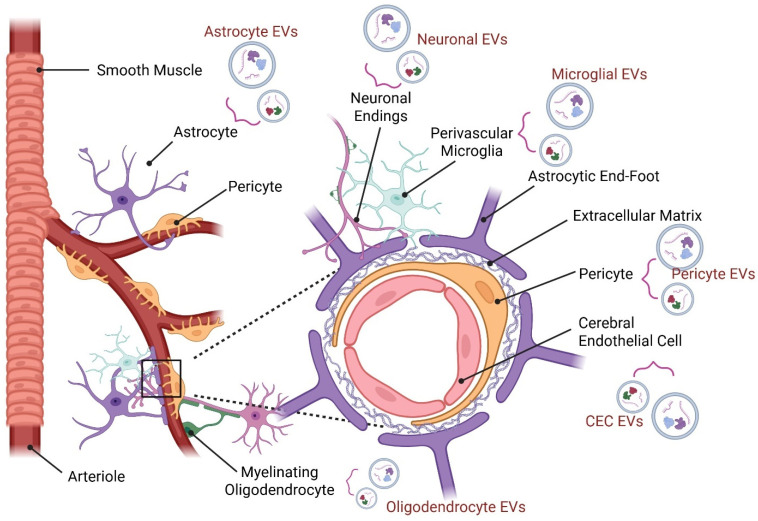
Structure of the NVU. Illustration of the NVU, highlighting key components including CECs, pericytes, astrocytic end-feet, microglia, and neurons. These cells are embedded within the extracellular matrix and closely interact to maintain brain homeostasis. EVs released by these NVU components mediate communication both within the NVU and with surrounding brain regions. Figure was Created in Biorender (https://www.biorender.com/).

As a leading cause of disability worldwide, stroke induces complex pathophysiological changes that disrupt the neurovascular unit (NVU), a group of closely interacting cells including neurons, astrocytes, cerebral endothelial cells (CECs), microglia, pericytes, and oligodendrocytes (Figure 1) [1]. The NVU provides the central nervous system (CNS) with the ability to maintain brain homeostasis and satisfactory oxygen and nutrient levels through local regulation of cerebral blood flow, known as neurovascular coupling [2,3]. Importantly, the NVU regulates the blood–brain barrier (BBB) that serves to protect the brain, filter harmful compounds between the brain and bloodstream, and supply brain tissue with nutrients [4]. The acute onset and rapid progression of ischemic injury often limit the effectiveness of current interventions. Clinically, tissue plasminogen activator (tPA) and endovascular thrombectomy (EVT) are used to restore blood flow and salvage viable brain tissue. However, tPA is constrained by a narrow therapeutic window (typically within 4.5 h) and an elevated risk of hemorrhagic transformation, while EVT, though effective up to 24 h in select patients, requires specialized equipment and expertise, limiting accessibility [5,6]. Full neurological recovery in ischemic stroke patients treated with tPA or EVT is not guaranteed, and most of these patients retain neurological deficits [7,8]. These challenges highlight the urgent need for adjunctive therapies that promote early perfusion, limit secondary injury, and support long-term recovery [9]. In this context, stroke treatment paradigms are shifting from a traditional neuroprotective focus, aimed at minimizing initial tissue damage, to one of neurorestoration, which emphasizes functional recovery through repair, neurovascular remodeling, and regeneration [10].

**Figure 2:**
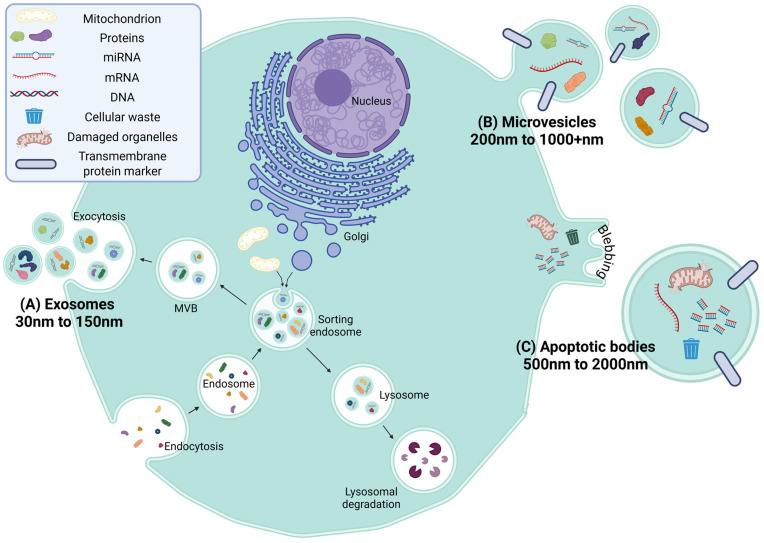
Schematic of EV Biogenesis. This diagram illustrates the formation and release of the three major EV subtypes: (**A**) Exosomes (30–150 nm) are generated through the endosomal pathway, where early endosomes mature into multivesicular bodies (MVBs) that either fuse with lysosomes for degradation or with the plasma membrane to release intraluminal vesicles (ILVs) as exosomes via exocytosis. (**B**) Microvesicles (200–1000+ nm) form by direct outward budding of the plasma membrane. (**C**) Apoptotic bodies (500–2000 nm) are released during programmed cell death through membrane blebbing. All EV types can carry diverse molecular cargo, including proteins, miRNAs, mRNAs, DNAs, lipids, cellular waste, damaged organelles, and transmembrane proteins, enabling them to mediate intercellular communication and influence recipient cell function. Figure was Created in Biorender (https://www.biorender.com/).

Extracellular vesicles (EVs) are small, membrane-bound entities secreted by nearly all cell types [11]. EVs play a key role in intercellular communication by transporting a variety of bioactive cargo—such as DNAs, RNAs, microRNAs (miRNAs), lipids, metabolites, and cytosolic or membrane-bound proteins—encapsulated within a lipid bilayer [12]. Their ability to cross biological barriers, including the BBB, and their involvement in diverse physiological and pathological processes have made them a major focus of biomedical research [13]. EVs are broadly categorized into three main subtypes based on their biogenesis: (1) exosomes (30–150 nm), formed through endosomal maturation and multivesicular body (MVB) fusion with the plasma membrane; (2) microvesicles (100–1000 nm), which bud directly from the plasma membrane; and (3) apoptotic bodies (500–2000 nm), released during programmed cell death [14] (Figure 2). Each subtype carries distinct cargo and may exhibit different functional roles in health and disease.

Numerous reviews have examined EVs as mediators of intercellular communication in neurologic disease, including their roles in stroke pathology, neuroinflammation, and regenerative signaling. Prior work has largely focused on EV biogenesis, cargo profiling, or individual cell-type contributions, often emphasizing neuroprotection in the acute phase of injury [9,15,16]. However, less attention has been given to EVs derived from distinct NVU cell populations and how they coordinate temporally to support neurorestorative processes following stroke. This review addresses this gap by elucidating how NVU-derived EV signaling across injury phases, communicate with resident NVU cell types and influence vascular stabilization, inflammation resolution, angiogenesis–neurogenesis coupling, white matter repair, and synaptic remodeling. By framing EVs as dynamic, phase-dependent modulators of post-stroke repair rather than solely acute neuroprotective agents, this review highlights emerging mechanisms of neurorestoration and identifies key translational challenges and opportunities for EV-based therapeutic strategies.

### Literature Search Strategy

Literature for this narrative review was identified through searches of PubMed and Google Scholar. Searches focused primarily on studies published between 2010 and 2025, with limited inclusion of earlier studies where relevant. Search terms were tailored to each major topic area and included combinations of keywords such as “extracellular vesicles,” “stroke,” “ischemic stroke,” “neurovascular unit,” “cerebral endothelial cells,” “astrocytes,” “microglia,” “angiogenesis,” “neurogenesis,” “oligodendrogenesis,” and “synaptic plasticity,” using Boolean operators (e.g., “stroke AND extracellular vesicles,” “cerebral endothelial cells AND extracellular vesicles”). In accordance with the 2018 MISEV guidelines, studies were preferentially included if they reported (i) EV isolation using ultracentrifugation, size-exclusion chromatography, precipitation-based kits, or comparable validated methods; (ii) particle sizing via nanoparticle tracking analysis (NTA), dynamic light scattering, or electron microscopy; and (iii) molecular characterization of canonical EV markers (e.g., CD9, CD63, CD81, Alix, TSG101) by Western blot or equivalent techniques. For studies published prior to 2018, similar minimal criteria were applied to determine inclusion, emphasizing physical characterization and at least one positive EV-associated marker [12]. Studies lacking EV characterization or relying solely on circulating vesicle measurements without isolation were excluded from mechanistic interpretation. Review articles and non-stroke models were included selectively to provide biological context but were not weighted as primary evidence for EV-mediated effects in ischemic stroke. Non-stroke studies were included only when the reported mechanisms were directly applicable to stroke pathology.

## 2. Cell Type-Specific EVs in the Structure and Function of the NVU

This section will review the various cell types of the NVU and highlight known mechanisms of how their EVs maintain homeostasis of CNS functions. In this section, we primarily focus on EVs released from the classical NVU components—neurons, CECs, astrocytes, and pericytes—as these represent the core structural and functional units governing neurovascular homeostasis. While microglia and oligodendrocytes are integral components of the broader NVU and are profoundly influenced by vascular and endothelial changes, their EV signaling roles are discussed primarily in the context of injury and repair rather than baseline homeostasis.

### 2.1. Neuron-Derived EVs

Neurons within the NVU play a critical role in maintaining brain homeostasis through coordinated interactions with astrocytes, microglia, CECs, and pericytes to regulate cerebral blood flow, synaptic signaling, and BBB integrity [17]. Under physiological conditions, neuron-derived EVs represent one component of this intercellular communication network and have been implicated in baseline signaling processes within the healthy brain.

Primary studies have demonstrated that neuron-derived EVs contain synaptic-associated proteins, including brain derived neurotrophic factor (BDNF), synaptophysin, and postsynaptic density protein 95 (PSD-95), consistent with a role in normal synaptic organization and neuron–glia communication [18]. Additional evidence confirms that neuron-derived EVs are enriched in synaptic and membrane-associated proteins, including synaptophysin and flotillin-1, consistent with their neuronal origin [18]. Moreover, EVs released from cortical neurons are internalized by recipient neurons and modulate spontaneous neuronal activity, highlighting a role for neuron-derived EVs in inter-neuronal communication and network regulation [19].

At baseline, neuron-derived EVs have been reported to contain bioactive cargo implicated in synaptic organization and plasticity, including adhesion proteins such as L1 adhesion molecule (L1CAM), synaptic components like α-Amino-3-hydroxy-5-methyl-4-isoxazolepropionic acid (AMPA) receptor subunits, and neuronal miRNAs (e.g., miR-124a). Heat shock proteins, including heat shock cognate protein 70 (Hsc70), have also been reported in neuron-derived EV preparations, suggesting potential roles in intercellular stress signaling and homeostatic communication across neural cell types [20,21]. Collectively, these observations position neuron-derived EVs as contributors to physiological signaling processes that support neuronal stability and synaptic integrity within the NVU.

### 2.2. Cerebral Endothelial Cell (CEC)-Derived EVs

CECs are core structural components of the NVU, forming a continuous monolayer along cerebral microvessels that underlies BBB integrity. These cells are interconnected by specialized tight junction complexes composed of zonula occludens-1 (ZO-1), claudins (-1, -3, -5, -12), and occludin, which restrict paracellular permeability and preserve brain homeostasis [22]. Through coordinated interactions with astrocytes, pericytes, neurons, and microglia, CECs contribute to the regulation of cerebral blood flow, participate in neurovascular signaling within the NVU, and dynamically respond to physiological stimuli within the NVU [23].

Under non-pathological conditions, CEC-derived EVs represent one component of endothelial communication within the NVU, contributing to signaling at the vascular-neural interface; however, there are limited direct mechanistic demonstrations of this interaction under healthy conditions. Reviews of NVU-derived EVs indicate that endothelial EVs carry heterogeneous molecular cargo reflective of their parent cell phenotype, including membrane-associated proteins, lipids, and regulatory RNAs, consistent with roles in intercellular signaling and phenotypic modulation, rather than serving as structural components of the barrier itself [24,25]. Although precise functional contributions of CEC-derived EVs in the healthy brain remain incompletely defined, their release is thought to contribute to baseline communication between vascular and parenchymal cell populations within the NVU.

### 2.3. Astrocyte-Derived EVs

Astrocytes are abundant glial cells within the NVU and are essential for maintaining CNS homeostasis through metabolic support, regulation of synaptic activity, and coordination of neurovascular responses. Through extensive contacts with synapses and cerebral microvessels, astrocytes help couple neuronal activity to vascular function and support BBB integrity via end-foot interactions with the cerebral endothelium and regulation of barrier-associated pathways [26,27,28]. In addition, astrocytes participate in glia-immune crosstalk, as microglia-derived cytokine signaling can induce reactive astrocyte phenotypes, highlighting how inflammatory cues can reshape astrocyte behavior within the CNS milieu [29].

Astrocytes also communicate with surrounding NVU cell types through EV release, and existing reviews describe them as heterogeneous vesicle populations whose protein, lipid, and RNA cargo reflect the functional state of the parent astrocyte [30]. In contrast to CEC-derived EVs, astrocyte-derived EVs are frequently discussed in the context of metabolic support, stress buffering, and homeostatic signaling within the neural parenchyma, consistent with their established roles in neuronal regulation and homeostatic support [24]. As with CEC-derived EVs, current understanding of astrocyte-derived EV function is largely based on injury and disease models. Under physiological conditions, astrocyte-derived EVs appear to participate in baseline intercellular signaling within the NVU, setting the stage for later discussion of how their profiles and functions are altered following ischemic injury and during neurorestoration.

### 2.4. Pericyte-Derived EVs

Pericytes are integral components of the NVU, closely associated with CECs and astrocytic end-feet, where they contribute to BBB stability, regulation of cerebral blood flow, and vascular homeostasis [31,32]. Through direct physical contact and paracrine signaling, pericytes influence endothelial permeability, microvascular tone, and structural maintenance of the cerebral microvasculature under physiological conditions.

Although pericytes are well recognized for their roles in angiogenic signaling, immune surveillance, and neurovascular remodeling, direct characterization of pericyte-derived EVs under strictly non-pathological conditions remains limited. Available evidence suggests that pericytes, like other NVU cell types, constitutively release EVs as part of their intercellular communication repertoire, consistent with their role in coordinating vascular and parenchymal interactions within the healthy brain [31].

Given their close association with brain capillaries and their established roles in regulating microvascular function, pericyte-derived EVs are well positioned to contribute to baseline signaling that supports endothelial state and local vascular regulation, likely contributing to endothelial signaling and microvascular regulation rather than serving as structural barrier elements. This limited understanding of pericyte EV function in physiological states underscores an important gap in the field and provides critical context for interpreting the expanded and altered roles of pericyte-derived EVs following ischemic injury and during neurorestorative processes.

These insights highlight NVU-derived EVs as vital contributors to baseline intercellular communication within the healthy brain. While the precise physiological functions of EVs vary by cell type and remain incompletely defined, their consistent presence across NVU components supports a role in maintaining neurovascular signaling homeostasis. Importantly, this baseline framework provides essential context for understanding how EV profiles, cargo, and functions are dynamically altered following ischemic injury and during subsequent neurorestorative processes, which are discussed in the following sections.

## 3. NVU-Derived EVs in Intercellular Communication Following Acute Ischemic Stroke

During ischemic stroke, the abrupt loss of cerebral blood flow initiates energy failure, excitotoxic cascades, oxidative stress, and secondary neuroinflammation, collectively disrupting NVU homeostasis [33]. The acute phase of stroke is characterized by a complex interplay of pathological processes, including ionic imbalance, mitochondrial dysfunction, inflammatory signaling, and programmed cell death, alongside the activation of endogenous protective responses within surviving tissue [34,35,36]. In this section, “acute” refers to processes occurring prior to structural tissue remodeling (angiogenesis, synaptogenesis, axonal sprouting) and instead encompasses signaling that determines cell survival, vascular integrity, and inflammatory tone within the first hours to ~3 days after ischemia.

The contribution of NVU-derived EVs evolves dynamically across the acute and chronic/recovery phases of ischemic injury, reflecting changes in cellular activation state and the shifting demands of the injured brain [17,37]. EV signaling following stroke reflects the physiological state of the parent cell and injury environment; vesicles released under acute ischemic stress may propagate inflammatory or degenerative cues, whereas vesicles generated during later recovery phases or following ischemic conditioning increasingly exhibit protective or adaptive properties [38]. Accordingly, NVU-derived EV communication during acute stroke can be functionally interpreted as comprising injury-propagating, injury-containing, and recovery-priming signaling pathways, a framework that guides the organization of the following sections. Beyond mechanistic contributions, circulating EV populations serve as early biomarkers of thrombo-inflammatory activity during acute stroke, with rapid detection of EV-associated cytokines and coagulation markers correlating with infarct burden and neurological severity. These findings position EVs not only as mediators of injury but also as potential diagnostic and prognostic tools in the hyperacute window.

The temporal progression of ischemic injury and recovery differs across experimental models and species. Rodent ischemic stroke models replicate key aspects of human stroke but do so at differing temporal scales, reflecting species-specific differences in injury progression and recovery [39]. Throughout this section, the acute phase is discussed in functional terms rather than strict temporal boundaries, with species and time points specified where relevant. Specifically, in experimental stroke models, the acute phase features metabolic failure, excitotoxicity, and inflammatory injury, followed by secondary injury, where emerging repair/remodeling processes overlap [33,40].

**Figure 3:**
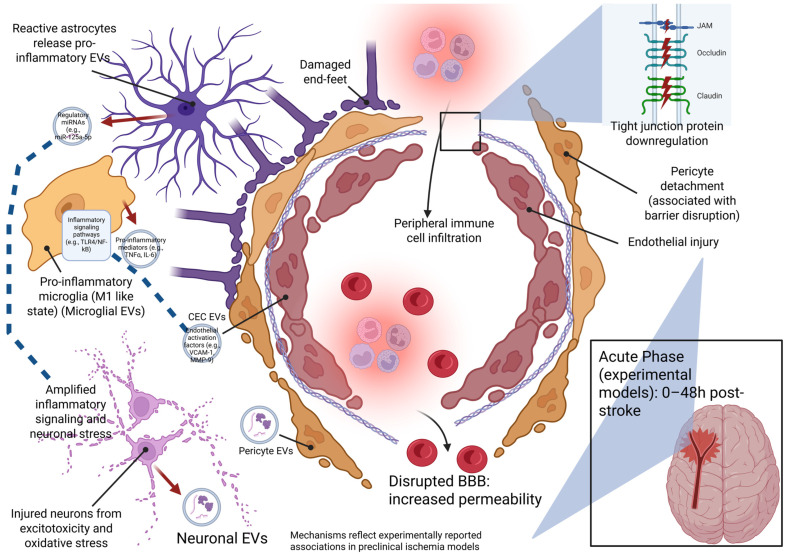
Acute Phase NVU-Derived Extracellular Vesicle Signaling Following Ischemic Stroke. Schematic representation of EV-mediated signaling within the NVU during the acute phase of ischemic stroke (primarily based on preclinical experimental models; ~0–48 h post-ischemia). Pro-inflammatory microglia (M1-like state), reactive astrocytes, injured neurons, endothelial cells, and pericytes release EVs containing regulatory microRNAs, inflammatory mediators, and endothelial activation factors that have been experimentally associated with blood–brain barrier (BBB) permeability changes, tight junction protein downregulation, endothelial activation, neuronal stress, and peripheral immune cell infiltration. Arrows represent reported associations in rodent ischemia models and do not imply temporally exclusive or universally causal mechanisms. EV effects are highly dependent on parent-cell activation state and local microenvironment. Figure was Created in Biorender (https://www.biorender.com/).

**Figure 4:**
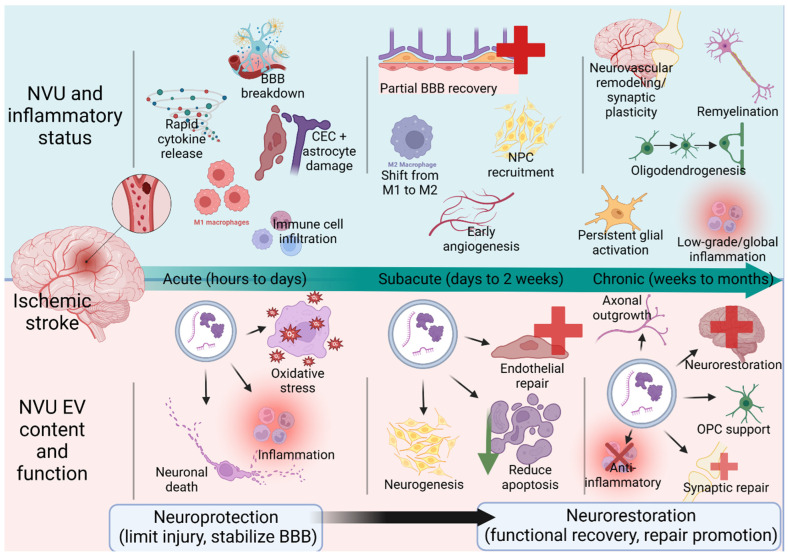
Temporal overview of ischemic stroke progression, illustrating evolving NVU dysfunction, inflammation, and EV signaling across ischemic stroke phases. This schematic summarizes phase-dependent changes in NVU dysfunction, inflammation, and EV-mediated signaling based primarily on preclinical rodent ischemia models (e.g., mouse and rat MCAO paradigms). Time ranges (acute: hours–days; subacute: days–~2 weeks; chronic: weeks–months) represent approximate experimental model frameworks rather than human clinical timelines. In the acute phase, NVU disruption triggers cytokine release and pro-inflammatory EV signaling associated with neuronal injury. During the subacute phase, inflammatory responses begin to resolve and EV signaling shifts toward reparative pathways including angiogenesis and cell survival. In the chronic phase, EVs contribute to synaptic plasticity, oligodendrogenesis, and circuit remodeling. Because stroke progression and recovery kinetics differ substantially across species, these temporal categories illustrate relative biological transitions rather than fixed translational timepoints. Arrows represent reported associations in rodent ischemia models and do not imply temporally exclusive or universally causal mechanisms. Plus signs indicate restorative outcomes. Figure was Created in Biorender (https://www.biorender.com/).

In this section, we examine how NVU-derived EVs participate in key aspects of acute stroke pathophysiology, including BBB disruption, thrombosis, neuronal survival within the ischemic penumbra, and the amplification or containment of inflammatory responses (summarized in Figure 3 and Figure 4 and Table 1 and Table 2).

### 3.1. Vascular and BBB Impairment

In the acute phase of ischemic stroke, disruption of cerebral vascular integrity and breakdown of the BBB represent early pathological events that exacerbate vasogenic edema, immune cell infiltration, and secondary tissue injury. BBB dysfunction arises from endothelial injury, loss of tight junction organization, and altered intercellular communication among NVU cell types, including CECs, astrocytes, and pericytes.

Evidence from endogenous EV release studies, conditioned media experiments, and exogenous EV administration models collectively suggests that EV-mediated signaling participates in early NVU communication during vascular stress; however, the degree of direct physiological contribution varies across cell types [46]. Under ischemic conditions, CEC-derived EVs have been reported to influence parenchymal cell responses through transfer of regulatory miRNAs, including miR-1290. These EVs are taken up by neurons in a caveolin-1 (Cav-1)-dependent manner and have been associated with reduced neuronal apoptosis following oxygen–glucose deprivation (OGD) and experimental stroke models [46]. While many mechanistic studies have focused on neuronal uptake and neuroprotection, these findings underscore the broader role of endothelial EVs in early NVU communication following ischemic insult. In mouse models of transient middle cerebral artery occlusion (tMCAO) with reperfusion (tMCAO/R), endothelial cell-derived exosomes were shown to modulate BBB permeability within the first 24–72 h after reperfusion. Specifically, in male C57BL/6J mice subjected to 90 min tMCAO followed by reperfusion, Sun et al. demonstrated that exosomes derived from cultured brain microvascular endothelial cells and administered during the acute post-ischemic period preserved BBB integrity by increasing tight junction protein expression (claudin-5, zonula ZO-1) and reducing Evans Blue and immunoglobulin G (IgG) leakage, with mechanistic involvement of platelet-derived growth factor-B/beta (PDGF/PDGFRβ) and angiopoiten-1/2- (Ang1/Ang2)–receptor tyrosine kinase (Tie2) signaling pathways [51]. These studies use exogenously administered endothelial EVs and therefore demonstrate signaling capacity rather than endogenous physiological production but collectively suggest endothelial EVs can modulate neuronal stress responses and vascular permeability within the first 24–72 h after ischemia.

In addition, evidence from ischemia-relevant experimental systems suggests that astrocyte-derived EVs can influence endothelial barrier stability under metabolic stress. In primary rodent astrocytes subjected to OGD, EVs collected following ischemia-like stress were shown to attenuate endothelial hyperpermeability by modulating Rho GTPase-activating protein 25 (ARHGAP25)-mediated/Wnt/β-catenin signaling, preserving tight junction organization in vivo [52]. Although demonstrated in rat models of intracerebral hemorrhage, these findings indicate that astrocyte EV cargo can regulate endothelial barrier pathways engaged during ischemia, although direct demonstration in ischemic stroke models remains limited.

Direct mechanistic evidence linking pericyte-derived EVs to BBB disruption during acute ischemic stroke remains limited. However, pericytes are anatomically integrated with cerebral microvessels and exert established control over endothelial stability and permeability. In a clinical study of human patients with acute ischemic stroke, circulating EV populations enriched for pericyte-associated markers were detected within 24 h of symptom onset and reflected dynamic microvascular signaling changes [54]. While these findings are correlative and do not establish causality, they support the presence of pericyte-associated EV signaling during acute vascular injury, but do not establish whether these vesicles actively modulate BBB permeability [55].

Evidence from rodent ischemia/reperfusion models, ischemia-mimetic in vitro systems, and early human clinical observations supports a role for NVU-derived EVs as modulators of intercellular communication during acute vascular and BBB impairment following stroke. Although the strength of mechanistic data varies across NVU cell types, particularly for pericyte-derived EVs, these studies establish a temporal, species-aware framework in which EV-mediated signaling contributes to early endothelial dysfunction prior to the initiation of reparative and neurorestorative processes.

### 3.2. Thrombosis

Thrombosis is a central pathological feature of acute ischemic stroke and arises through coordinated platelet activation, fibrin deposition, endothelial activation, and inflammatory signaling. Thrombi may arise from large-artery atherosclerotic disease, in which unstable plaques can generate thromboembolic material that travels distally or lead to in situ thrombosis at the lesion site [56]. In both settings, interactions among platelets, leukocytes, endothelial cells, and circulating coagulation factors drive clot propagation and vascular occlusion [57]. Importantly, thrombi retrieved from patients with acute ischemic stroke are highly heterogeneous in composition, containing variable proportions of fibrin, platelets, neutrophil extracellular traps (NETs), and von Willebrand factor (vWF), each of which contributes to thrombus stability, resistance to reperfusion therapies, and downstream vascular injury [58,59]. Fibrin-rich thrombi exhibit increased resistance to intravenous thrombolysis and mechanical thrombectomy, often requiring multiple recanalization attempts and correlating with poorer clinical outcomes [60,61]. Notably, many pro-coagulant EV populations detected in circulation during stroke likely originate from both intravascular NVU cells and peripheral immune cells, complicating attribution of specific functional effects to brain-derived vesicles.

Emerging evidence indicates EVs derived from NVU and immune cell populations actively participate in thrombo-inflammatory signaling during acute ischemic stroke by linking vascular injury, coagulation, and endothelial dysfunction. In the acute phase, circulating EV populations isolated from stroke patients exhibit pro-coagulant and inflammatory profiles, consistent with increased matrix metalloproteinase-9 (MMP-9) activity and elevated interleukin-1β (IL-1β) and tumor necrosis factor-α (TNF-α) signaling observed in acute ischemic stroke [53]. This cargo profile is consistent with EV-mediated endothelial activation and engagement of coagulation pathways [62]. Transcriptomic analyses of EVs isolated from human plasma and thrombi collected during acute intervention further demonstrate enrichment of pathways related to coagulation, endothelial stress responses, and inflammatory activation, supporting a mechanistic role for EVs in shaping thrombotic microenvironments [53]. Because thrombi incorporate endothelial fragments, platelets, leukocytes, and NETs, these EVs likely represent a composite vascular microenvironment rather than a single cellular source.

Within the NVU, direct experimental evidence demonstrates that clot-associated EVs can act as effectors of endothelial injury. Small EVs isolated from human ischemic stroke clots obtained at the time of mechanical thrombectomy significantly impaired human CEC viability, reduced angiogenic capacity, and increased endothelial permeability in vitro [63]. These clot-derived EVs exhibited a pro-coagulant surface profile characterized by elevated phosphatidylserine and tissue factor, suggesting that EVs released directly from thrombi may propagate local microvascular thrombosis while exacerbating endothelial barrier disruption at the infarct site [63]. This finding provides direct mechanistic support for EVs as active participants of thrombus-associated vascular injury.

In addition to pathogenic roles, EVs may serve as early biomarkers of thrombo-inflammatory activity in acute stroke. In a clinical study using a mobile stroke unit, plasma-derived EVs obtained from human patients as early as approximately 36 min after symptom onset displayed distinct inflammatory marker profiles, including elevated EV-associated interleukin-6 (IL-6), TNF-α, monocyte chemoattractant protein-1 (MCP-1), and interleukin-1 receptor antagonist (IL-1RA) [64]. Levels of these EV-associated markers correlated with infarct size and neurological deficit severity, underscoring the rapid involvement of EV signaling in acute stroke pathophysiology and supporting their potential utility as early biomarkers of thrombo-inflammatory burden.

These findings support a model in which EVs participate in thrombus-associated signaling environments during acute ischemic stroke by integrating pro-coagulant surface features with inflammatory and matrix-modifying cargo that promotes endothelial activation, thrombus heterogeneity, and resistance to reperfusion therapies. While direct mechanistic evidence linking NVU-derived EVs to thrombus initiation remains limited, existing data indicate that EV-mediated crosstalk between the injured NVU, immune system, and thrombotic milieu plays a meaningful role in shaping vascular dysfunction during the acute phase of stroke. This positions EVs as both mechanistically relevant biomarkers and potential modulators of thrombo-inflammatory signaling in acute ischemic injury.

### 3.3. Neuroprotective Functions

Preservation of the ischemic penumbra, the region of hypoperfused yet viable tissue surrounding the infarct core, is a central determinant of neurological recovery following acute ischemic stroke. Penumbral neurons are exposed to metabolic stress, excitotoxic signaling, oxidative damage, and inflammatory-mediated injury, yet remain salvageable within a defined temporal window [41]. Increasing evidence indicates that EVs released from NVU and neural cell populations participate in intercellular signaling processes that influence neuronal stress responses, plasticity, and survival pathways in the nervous system [65]. In this context, “neuroprotective” refers to preservation of stressed but viable cells rather than long-term tissue remodeling, which is addressed in later sections.

CEC-derived EVs have been shown to directly promote axonal growth following ischemia–reperfusion injury. In a mouse tMCAO model, EVs isolated from ischemic brain microvascular endothelial cells enhanced axonal growth compared with EVs from non-ischemic endothelium, in part through miRNA-mediated suppression of intrinsic axonal growth inhibitors including phosphatase and tensin homolog (PTEN), Ras homolog family member A (RhoA), and semaphorin 6A (Sema6A) [42]. Beyond axonal remodeling, CEC-derived sEVs also enhance neurovascular stability in acute stroke models by augmenting tPA-induced thrombolysis, improving downstream microvascular perfusion, reducing BBB leakage, and ultimately decreasing infarct volume and improving neurological outcomes [43]. These findings suggest that ischemia alters endothelial EV cargo in ways that are associated with enhanced axonal growth and activation of regenerative signaling pathways within peri-infarct tissue. These effects represent early activation of regenerative programs rather than immediate prevention of cell death.

Exogenously administered endothelial progenitor cell (EPC)-derived EVs, which are vascular-associated rather than parenchymal NVU components, also exert neuroprotective effects in rodent models of focal cerebral ischemia. When administered 24 h after stroke onset, EPC derived EVs reduced infarct volume, neuronal apoptosis, and tissue degeneration while enhancing neurological recovery [44]. These effects were associated with increased expression of endothelial and vascular survival markers such as platelet endothelial cell adhesion molecule-1 (PECAM-1) and vascular endothelial growth factor (VEGF) and support a role for EPC-derived EVs in stabilizing the ischemic microenvironment during the penumbral phase. Although EPCs are not classical resident NVU structural cell types, EPC-derived EVs provide a model of vascular-associated EV signaling that can stabilize the ischemic microenvironment. Consistent with this concept, endogenous EPC mobilization and higher circulating EPC levels after acute ischemic stroke have been associated with improved neurological and functional outcomes in clinical studies [45].

Astrocyte-derived EVs represent another major source of neuroprotective signaling following ischemic injury. Multiple studies in rodent ischemia or OGD models demonstrate that astrocyte-derived EVs delivered within hours to ~24 h after injury are associated with reduced neuronal apoptosis [48,66] and inflammasome-associated pyroptotic signaling [67,68] and attenuate inflammatory pathway activation. Astrocyte-derived EV cargo enriched in miR-378a-5p, miR-34c, miR-628, and miR-190b have been associated with suppression of pro-death signaling pathways including NOD-like receptor family pyrin domain-containing 3 (NLRP3) inflammasome activation, toll-like receptor-7 (TLR7) signaling, nuclear factor kappa B (NF-κB)/MAPK cascades, and autophagy-related pathways, thereby limiting neuronal loss and promoting a neuroprotective microglial phenotype [42,48,49,66,67,68,69,70,71]. These studies consistently report treatment timepoints ranging from hours to 24 h post-ischemia, aligning with the established therapeutic window for penumbral rescue.

Neuron-derived EVs may also influence the survival of stressed but viable neurons within the penumbra. In a mouse middle cerebral artery occlusion (MCAO) model, intracerebroventricular administration of a miRNA-98 agomir 3 days prior to ischemia suppressed expression of platelet-activating factor receptor (PAFR), attenuating microglial engulfment of peri-infarct neurons and improving functional outcomes 1–3 days after ischemia while reducing infarct volume [69]. Because treatment occurred before ischemia, this model reflects EV-mediated conditioning of neuroimmune signaling rather than endogenous acute release, but it identifies a pathway through which neuronal EV cargo could limit secondary neuronal loss once present in the injury environment. Additional neuron-derived EV populations have been implicated in modulation of neuroinflammatory signaling, including suppression of pro-inflammatory cytokine expression and promotion of anti-inflammatory microglial phenotypes, suggesting immune regulation as a mechanism contributing to penumbral preservation [70].

Mechanistically, neuronal uptake of endothelial cell-derived EVs is mediated by Cav-1–dependent endocytosis. In OGD-treated neurons and in vivo middle cerebral artery occlusion (MCAO) models, Cav-1 expression was significantly upregulated [46]. Small interfering RNA-mediated knockdown of Cav-1 reduced EV internalization and abolished EV-mediated attenuation of neuronal apoptosis, identifying Cav-1 as a critical regulator of EV-based neuroprotective signaling during acute ischemic injury [46]. This uptake mechanism provides a shared regulatory axis through which EVs from multiple NVU sources converge on vulnerable neuronal populations.

Collectively, these findings indicate that EV signaling from multiple NVU and vascular-associated sources can limit secondary neuronal loss by modulating apoptosis, inflammatory activation, and phagocytic clearance of stressed neurons. Rather than acting as a single coordinated protective program, the available evidence supports parallel cell-type-specific signaling pathways that bias the acute injury environment toward tissue preservation, thereby increasing the amount of salvageable substrate available for later neurorestorative processes.

### 3.4. Inflammatory Response

Acute neuroinflammation is a defining early response to ischemic stroke and is initiated within minutes to hours of cerebral ischemia through coordinated activation of resident immune cells, endothelial dysfunction, and rapid recruitment of peripheral leukocytes. In both experimental models and human patients, early inflammatory signaling involves microglial activation, cytokine release, oxidative stress, and disruption of BBB integrity, which together exacerbate neuronal injury and secondary tissue damage [72,73]. While early inflammatory responses contribute to debris clearance and tissue remodeling, excessive or prolonged activation of immune pathways promotes neuronal death, edema, and chronic neuroinflammation that impairs functional recovery [40].

Microglia serve as the primary initiators of post-stroke neuroinflammation, and increasing evidence indicates that microglia-derived EVs may be critical mediators of inflammatory signal propagation following ischemic injury. Under pathological conditions, EVs released from classically activated (M1-like) microglia are enriched in pro-inflammatory cytokines, damage-associated proteins, and regulatory RNAs that amplify neuroinflammation and neuronal damage [50,72,73]. These EVs, when released under inflammatory conditions, have been reported to contain regulatory RNAs and signaling molecules capable of activating NF-κB-dependent inflammatory pathways and amplifying glial and endothelial activation. In experimental stroke mouse models, M1 microglia-derived exosomes enriched in circSTRN3 have been shown to exacerbate ischemic injury and have been associated with shifts toward neurotoxic A1-like astrocytic phenotypes through sponging of miR-331-5p and activation of MAVS-dependent NF-κB signaling, resulting in increased neuronal apoptosis during the early post-ischemic period (24–72 h after MCAO/reperfusion) [47]. Environmental and metabolic stressors further shape inflammatory EV cargo, as microglial exposure to saturated fatty acids or extracellular ATP alters EV proteomic and RNA composition, enhancing inflammatory signaling in recipient astrocytes and neurons [74,75].

In contrast, alternatively activated (M2-like) microglia release EVs that bias the inflammatory environment toward resolution. In preclinical murine tMCAO models, M2 microglia-derived EVs administered during the early post-ischemic period suppress inflammasome activation and reduce tissue injury, in part through delivery of miR-135a-5p targeting the TXNIP/NLRP3 axis [76]. These findings indicate that M2 microglial EVs can actively restrain excessive innate immune activation within the acute injury environment.

EVs released from other NVU components also contribute to inflammatory amplification or containment following stroke. Astrocytes interact dynamically with microglia, shifting between neurotoxic (A1-like) and neuroprotective (A2-like) states in response to cytokines such as interleukin-1α (IL-1α), TNF-α, and complement component 1q (C1q) [29]. In addition, when exposed to key mediators of glial activation and glial damage, TNF-α and IL1β, primary rat astrocytes released EVs that were enriched with miRNAs that target proteins involved in neurotrophic signaling. These miRNAs, miR-125a-5p and miR-16-5p, alter neuronal activity-related protein expression by regulating the translational expression of proteins controlling programs essential for synaptic stability and neuronal excitability [77]. Astrocyte-derived EVs reflect these activation states and can modulate inflammatory signaling by transferring cytokines, miRNAs, and regulatory proteins that influence neuronal survival and synaptic integrity [78]. Neuron-derived EVs released under stress conditions have been shown to carry regulatory RNAs and proteins capable of modulating microglial activation and endothelial responses, associated with BBB dysfunction and neuronal vulnerability when inflammation is sustained [79].

Although pericyte-derived EVs have been less extensively studied in ischemic stroke, evidence from related CNS injury models indicates that these vesicles can influence inflammatory and oxidative stress pathways relevant to stroke pathology. Pericyte-derived EVs carrying miR-210 improve mitochondrial function, reduce lipid peroxidation, and reinforce endothelial barrier stability through JAK1/STAT3 signaling in models of spinal cord injury, indicating pathways that could be relevant to cerebral ischemia but remain unconfirmed in stroke models [80].

Collectively, NVU-derived EVs participate in post-stroke neuroinflammatory communication networks, amplifying injury when released from pro-inflammatory cellular states and constraining inflammation when originating from resolving phenotypes. Rather than acting as dedicated inflammatory mediators, these vesicles reflect the activation state of their parent cells and distribute cytokines, regulatory RNAs, and lipids that influence neighboring cell behavior. This positions EV signaling as a mechanism through which local cellular states are broadcast across the injured NVU during the acute phase of stroke.

## 4. NVU-Derived EVs in Neurorestoration After Stroke

In contrast to their early biomarker and injury-associated roles, EV populations present in brain during later phases of stroke are increasingly investigated as therapeutic vectors capable of promoting angiogenesis, WM, and synaptic remodeling. Following the acute injury response, ischemic stroke transitions into the chronic/recovery phase, which is characterized by endogenous repair and remodeling processes within the NVU [81]. During these stages, surviving neural and vascular cells engage in coordinated programs aimed at restoring tissue integrity, reestablishing network connectivity, and supporting long-term functional recovery [82]. The contribution of NVU-derived EVs continues to evolve during post-stroke recovery, reflecting changes in cellular activation state and the shifting priorities of the injured brain. In contrast to the acute phase, EVs released during later stages increasingly support reparative and neurorestorative processes, including vascular stabilization, neural survival, and structural remodeling [83,84].

**Figure 5:**
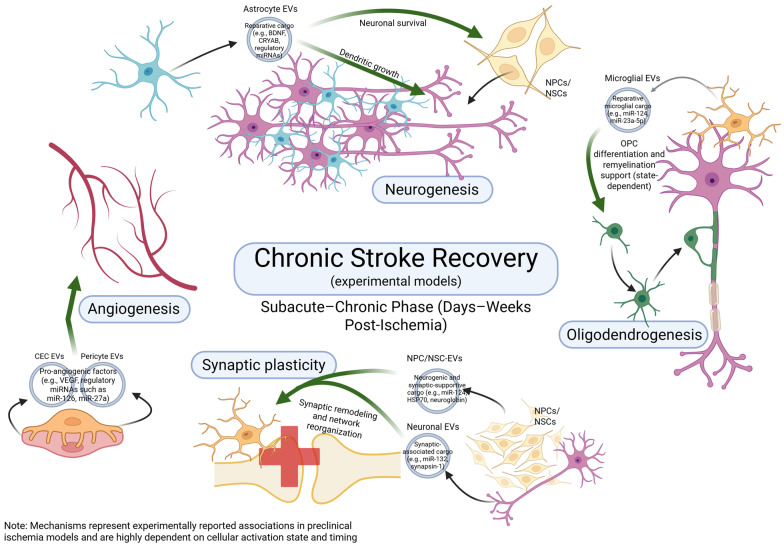
EV-Mediated Neurorestoration During the Chronic Phase of Ischemic Stroke. Schematic representation of EV-associated signaling within the NVU during the chronic phase of ischemic stroke (primarily based on preclinical experimental models; days–weeks post-ischemia). Endothelial, pericyte, astrocyte, microglial, neuronal, and neural progenitor/stem cell-derived EVs have been experimentally associated with angiogenesis, neurogenesis, oligodendrogenesis, and synaptic remodeling. Reported EV cargo includes pro-angiogenic factors (e.g., VEGF and regulatory microRNAs), reparative astrocytic and microglial mediators (e.g., BDNF, CRYAB, miR-124, miR-23a-5p), and synaptic-supportive cargo (e.g., miR-132, synapsin-1, HSP70, neuroglobin). Green arrows indicate restorative processes such as neuronal survival, dendritic growth, OPC differentiation and remyelination support, and synaptic remodeling/network reorganization. Mechanisms illustrated reflect experimentally reported associations in preclinical ischemia models and are highly dependent on cellular activation state, timing, and microenvironmental context rather than representing temporally exclusive or universally causal pathways. Arrows represent reported associations in rodent ischemia models and do not imply temporally exclusive or universally causal mechanisms. Figure was Created in Biorender (https://www.biorender.com/).

As in the acute setting, the temporal progression of recovery differs across experimental models and species. In rodent models, the chronic/recovery phase typically unfolds over days to weeks, whereas in human stroke these processes extend over weeks to months [85]. Throughout this section, the chronic/recovery phase is discussed in functional terms rather than strict temporal boundaries, with species and treatment timepoints specified where relevant. In this section, we examine how NVU-derived EVs contribute to key mechanisms of post-stroke neurorestoration, including angiogenesis, axonal remodeling, synaptic plasticity, oligodendrogenesis, and WM repair (summarized in Figure 4 and Figure 5 and Table 2 and Table 3).

### 4.1. Angiogenesis and Vascular Remodeling

Angiogenesis and vascular remodeling are widely recognized contributors to post-stroke neurorestoration, enabling restoration of cerebral perfusion, metabolic support, and neurovascular coupling within peri-infarct tissue [86]. Following ischemic stroke, neovascularization occurs primarily in peri-infarct regions over days to weeks and has been associated with neuronal survival, structural remodeling, and improved long-term functional outcomes [111,112]. While angiogenic responses can be detected within the early phase, effective vascular remodeling is driven by coordinated signaling between endothelial cells, pericytes, astrocytes, immune cells, and neural elements within the NVU [87].

EVs released from NVU components have emerged as key mediators of post-stroke angiogenesis, acting as vehicles for delivery of pro-angiogenic miRNAs, growth factors, and regulatory proteins that shape endothelial behavior and vessel maturation. In rat models of embolic and tMCAO, administration of CEC-derived small EVs in combination with tPA significantly reduced infarct volume, enhanced recanalization, increased cerebral blood flow, and reduced blood–brain barrier leakage [87]. These effects were associated with suppression of pro-thrombotic and pro-inflammatory signaling networks within the NVU, supporting a role for endothelial-derived EVs in limiting acute neurovascular damage. EPC-derived EVs further contribute to post-stroke angiogenesis, although EPCs are vascular-associated cells rather than classical structural NVU components. In rodent models of focal cerebral ischemia, EPC-derived EVs administered during the chronic/recovery phase (7 days post-stroke) enhanced endothelial cell proliferation, migration, and tube formation in vitro, increased cortical microvessel density in vivo, reduced infarct volume, and improved neurological outcomes. These pro-angiogenic effects were linked to EV cargo enriched in pro-angiogenic miRNAs, including miR-126, which modulated the phosphoinositide 3-kinase (PI3K)/Akt pathway [92]. Importantly, EPC-derived EVs modified by ischemic or pharmacological conditioning exhibit enhanced reparative efficacy, underscoring the influence of parent cell state on EV function [93].

Beyond promoting vascular expansion alone, emerging evidence indicates that endothelial-derived EV signaling may also influence neural remodeling processes, suggesting that angiogenesis and axonal repair are coordinated during post-stroke recovery. Ischemia has been shown to reshape the miRNA cargo of endothelial-derived EVs, altering their regulatory influence on recipient cells. Experimental stroke models demonstrate that ischemia-conditioned endothelial EVs carry pro-regenerative miRNAs capable of targeting pathways that restrict axonal growth, including PTEN- and RhoA-associated signaling cascades [98]. These findings suggest that endothelial EVs may contribute to the coordinated remodeling of vascular and neural compartments during recovery, reinforcing the functional coupling between angiogenesis and axonal repair. Pericyte-derived EVs represent a potential, though less extensively characterized, contributor to vascular remodeling within the NVU. While stroke-specific in vivo data remains limited, studies in ischemic conditioning and chronic vascular stress contexts indicate that pericyte-derived EVs can modulate endothelial signaling pathways involved in angiogenesis and vascular stabilization [99]. These findings suggest that pericyte-derived EVs may participate in endothelial adaptation under ischemic conditions. Supporting this concept, hypoxia-exposed placental pericytes release EVs enriched in pro-angiogenic factors that enhance endothelial tube formation and migration in vitro [100]. Although not derived from brain tissue, these data indicate that pericytes respond to hypoxic stress by modifying EV cargo in ways that promote endothelial remodeling. Collectively, these observations raise the possibility that pericyte-derived EVs contribute to microvascular stabilization and repair during the chronic/recovery phase of stroke recovery, a hypothesis that warrants further direct investigation.

Astrocyte-derived EVs also participate in angiogenic signaling after stroke, particularly within hypoxia-adapted environments. In rodent models of ischemia–reperfusion, intracerebral administration of astrocyte-derived EVs accelerates spontaneous functional recovery and enhances axonal structural remodeling within peri-infarct circuits suggesting that astrocyte-derived EVs may contribute to post-stroke tissue reorganization beyond their traditional roles in gliosis [113]. Similarly, microglia-derived EVs influence angiogenesis indirectly by modulating post-stroke inflammation and perivascular astrocytic AQP4 polarization. This was demonstrated in experimental stroke models, where hypoxia-conditioned microglial EVs reduce AQP4 depolarization, improve cerebrospinal fluid dynamics, attenuate periinfarct edema, and enhance cerebral perfusion [88,114]. By stabilizing the perivascular microenvironment, these EVs may create conditions that are supportive of effective vascular remodeling. Consistent with this context-dependent signaling, hypoxia-preconditioned microglia-derived EVs further enhance angiogenesis, repress apoptosis, and attenuate astrocytic aquaporin-4 (AQP4) depolarization in stroke models through TGF-β/Smad2/3 signaling, highlighting how EV cargo composition reflects microglial activation state and local oxygen availability [88,115]. Further extending this principle, engineered microglia-derived EVs containing miR-124 or miR-711 similarly attenuate neurodegeneration in preclinical CNS disease models by modulating metabolic and inflammatory pathways [116,117].

Beyond angiogenesis, endothelial-derived EVs further contribute to vascular remodeling by preserving blood–brain barrier integrity during repair. This was demonstrated in rodent stroke models, where endothelial EV treatment attenuated endothelial permeability and reduced matrix-degrading signaling pathways associated with vascular leakage [118]. These findings reinforce the concept that effective angiogenesis after stroke requires not only vessel formation but also coordinated barrier restoration.

Rather than acting as isolated pro-angiogenic signals, these EV populations appear to coordinate endothelial proliferation, vessel stabilization, barrier restoration, and neural remodeling through overlapping regulatory pathways shaped by parent-cell activation state. Collectively, EVs derived from endothelial cells, EPCs, pericytes, astrocytes, and microglia contribute to post-stroke angiogenesis and vascular remodeling through temporally regulated intercellular signaling. By integrating endothelial proliferation, vessel stabilization, blood–brain barrier restoration, and neurovascular coupling, EV-mediated communication supports coordinated vascular regeneration that underlies subsequent neuronal and WM repair. These properties support a role for NVU-derived EVs as modulators of neurovascular remodeling and potential candidates for therapeutic exploration, pending further validation across experimental models and dosing paradigms.

### 4.2. Neurogenesis and NPC Support

Adult neurogenesis is reactivated following ischemic stroke, predominantly within the subventricular zone (SVZ) and the subgranular zone (SGZ) of the hippocampus, where neural stem cells (NSCs) and neural progenitor cells (NPCs) generate new neurons in response to injury [119]. SVZ-derived neuroblasts can migrate toward peri-infarct regions and may contribute to tissue remodeling and functional recovery, whereas SGZ neurogenesis exhibits more context-dependent effects, with aberrant integration of newly generated neurons has been associated with cognitive dysfunction and post-stroke dementia in some settings [120]. Across models, the survival and functional integration of newly generated neurons remain limited, in part due to sustained inflammation, metabolic stress, and insufficient trophic support [109]. Neuroinflammatory tone critically shapes this process, with pro-inflammatory microglial signaling contributing to suppression of progenitor cell differentiation and integration, whereas anti-inflammatory and trophic microglial phenotypes appear to support neurogenesis and neuronal survival [89]. Epigenetic regulation, including DNA methylation, histone modification, and non-coding RNAs such as miRNAs such as miR-124 and members of the miR-17-92 cluster, further governs NSC fate decisions during post-stroke recovery [121].

Increasing evidence indicates that EVs derived from NVU and neural progenitor populations act as key mediators of post-stroke neurogenesis by supporting neural progenitor cell (NPC) proliferation, neuronal differentiation, and intercellular signaling within neurogenic niches. In rodent ischemic stroke models, NPC-derived EVs improve functional recovery even when endogenous neurogenesis is pharmacologically suppressed, indicating that EV-mediated signaling contributes to post-stroke functional recovery independently of de novo neuron production [122]. Induced neural progenitor cell-derived EVs (iNPC-EVs) similarly enhance post-stroke recovery in tMCAO mice, increasing Sox2^+^ and Ki67^+^ progenitor populations, elevating NeuN^+^ and DCX^+^ cell abundance, and reducing apoptotic and inflammatory markers including ionized calcium-binding adaptor molecule 1 (Iba1) and COX-2 [123]. These findings suggest that progenitor-derived EVs influence both NPC dynamics and the inflammatory microenvironment.

CEC-derived EVs also contribute to neurogenic regulation under conditions of vascular compromise. In aged diabetic rat models, CEC-derived small EVs isolated from healthy donors improved cognitive performance, enhanced neurogenesis, and restored cerebral vascular function following systemic administration. These effects were associated with EV uptake by NSCs within neurogenic regions, increased expression of miR-1 and miR-146a, and reduced MyD88 and thrombospondin-1 protein levels [124]. These data support a model in which endothelial EVs couple vascular stabilization with neurogenic support, particularly under conditions of vascular compromise.

Astrocyte-derived EVs represent another major source of neurogenic regulation under both physiological and pathological conditions. EVs released from astrocytes exposed to stress or injury carry miRNAs such as miR-9, miR-26a, and miR-34a that have been implicated in regulating neural progenitor proliferation and differentiation [110]. Under neuroprotective conditions, astrocyte-derived EVs contain trophic cargo including fibroblast growth factor (FGF-2), VEGF, apolipoprotein-D (Apo-D), and regulatory proteins that support neuronal survival, synaptic plasticity, and maturation [30]. In vitro studies using human cortical neurons further demonstrate that astrocyte-derived EVs enhance neuronal electrophysiological function, increasing excitability and action potential firing, consistent with a potential role in post-stroke neuronal maturation and circuit refinement [125]. EVs derived from neural progenitors also influence lineage specification and differentiation dynamics. Neural stem/progenitor cell (NSPC)-derived EVs promote astrocytic differentiation in a dose-dependent manner in vitro, accompanied by reduced nestin expression and increased GFAP-positive [126].

Beyond local effects, neuron-derived EVs have emerged as potential biomarkers of neurogenic activity. In human studies, alterations in doublecortin levels within neuron-derived EVs following electroconvulsive therapy, suggesting that neuronal EV signatures may reflect dynamic neurogenic states [127]. Mechanistically, iNPC-derived EVs promote wild-type NPC proliferation in part via ERK-dependent signaling pathways [128], while EVs derived from human induced pluripotent stem cell-derived NSCs enhance hippocampal neurogenesis and suppress inflammation in preclinical models, supporting their potential therapeutic relevance in models of brain injury [129].

Collectively, EVs derived from NPCs, NSCs, CECs, astrocytes, and neurons contribute to an interconnected signaling environment that shapes post-stroke neurogenesis by delivering pro-neurogenic miRNAs, trophic factors, and immunomodulatory cargo. By coupling progenitor proliferation and neuronal differentiation with inflammation control and synaptic maturation, this vesicle-mediated communication supports neural repair in experimental models of brain injury.

### 4.3. Oligodendrogenesis and Myelin Repair

Ischemic stroke induces significant oligodendrocyte and WM injury, resulting in demyelination, axonal instability, and impaired neural conduction that contribute to long-term functional deficits. Oligodendrocytes are particularly vulnerable to ischemic injury due to their high oxidative metabolic demand, elevated intracellular iron levels, low glutathione-mediated antioxidant capacity, and Ca^2+^-permeable glutamate receptor signaling, making them early targets of hypoxia–ischemia-induced damage [130]. WM injury can precede overt axonal degeneration, with experimental studies demonstrating early myelin loss within days of ischemic insult, followed by progressive axonal disruption that correlates with delayed functional recovery [131].

Despite this vulnerability, ischemic injury also triggers endogenous oligodendrocyte repair programs. Oligodendrocyte progenitor cells (OPCs) proliferate and migrate toward peri-infarct regions, where they differentiate into mature oligodendrocytes and may contribute to remyelination [132]. This process is influenced by signaling pathways, such as Sonic hedgehog, Wnt/β-catenin, PI3K/Akt, and STAT3, which collectively govern OPC survival, differentiation, and myelin formation [133]. Epigenetic mechanisms further shape oligodendrogenesis, including miRNAs (e.g., miR-17–92, miR-9) as well as histone deacetylases modulating lineage progression and myelin gene expression [106]. However, although OPC recruitment can be robust, their differentiation often remains incomplete, leading to prolonged myelin deficits and impaired WM repair [107].

The inflammatory microenvironment strongly influences oligodendrogenesis and remyelination efficiency. Acute inflammatory responses may support OPC recruitment, whereas sustained or dysregulated inflammation inhibits OPC maturation and promotes WM degeneration. TGFα is upregulated following ischemic stroke and preserves OPC and oligodendrocyte viability via STAT3 signaling, contributing to preservation of WM integrity after ischemia [101]. Conversely, chronic microglial activation and persistent pro-inflammatory cytokine signaling impair remyelination. Experimental modulation of the post-stroke environment, such as enriched housing conditions, is associated with reduced microglial activation, decreased IL-1β and IL-6 levels, improved WM integrity, and enhanced cognitive performance in rodent stroke models [102]. Additional injury mechanisms, including ferroptosis, which contributes to OPC loss and WM injury in hemorrhagic stroke models, underscore the need for strategies that both protect oligodendrocyte lineage cells and support their maturation [103].

Within this reparative landscape, microglia-derived EVs emerge as important regulators of oligodendrogenesis and myelin repair. Microglia-derived EVs exhibit context-dependent effects on remyelination. EVs released from pro-inflammatory microglia can impair OPC differentiation, in part by altering astrocyte responses within demyelinated lesions [104,134]. In contrast, EVs derived from interleukin-4 (IL-4)-stimulated or mesenchymal stem cell (MSC)-primed microglia promote OPC recruitment, differentiation, and WM repair [135]. These pro-regenerative EVs deliver signaling molecules including transmembrane tumor necrosis factor (TNF), which activates TNFR2 in OPCs and promotes their maturation, improving neurologic functional recovery following stroke [135]. Microglial EVs enriched in miR-23a-5p further support OPC proliferation and survival by enhancing OPC differentiation and remyelination-associated gene expression, resulting in enhanced remyelination and improved behavioral outcomes [94]. Independent evidence also shows that M2-derived EVs enriched in miR-23a-5p promote oligodendrocyte precursor proliferation and white matter repair after ischemic insult, contributing to neurological recovery [133].

In parallel, lipidomic analyses demonstrate that microglia- and macrophage-derived EVs carry bioactive endocannabinoids, including anandamide and 2-arachidonoylglycerol, which regulate OPC maturation; pharmacological blockade of cannabinoid receptors 1 and 2 abolishes EV-induced oligodendrogenesis, confirming a functional role for EV lipid cargo in remyelination [95]. These findings indicate that EV composition coordinates RNA- and lipid-mediated signaling to shape repair outcomes. Endothelial cell-derived EVs represent an additional source of pro-oligodendrogenic signals. EVs derived from CECs enhance OPC survival, proliferation, and motility, effects associated with activation of Akt- and Src-dependent pathways in oligovascular signaling [136,137]. These EVs reduce apoptotic OPC death and support WM integrity, functions that are particularly relevant in the ischemic microvasculature.

Astrocyte-derived EVs influence OPC differentiation and myelin-associated processes by delivering proteins and regulatory RNAs that influence OPC differentiation and myelination. Astrocyte-derived EV cargo includes growth factors such as FGF-2 and VEGF, as well as Apo-D, a lipid-binding protein implicated in myelin maintenance, supporting oligodendrocyte survival and maturation [138]. However, astrocyte aging and disease-associated phenotypic shifts attenuate these supportive effects. EVs released from aged astrocytes lose the capacity to support OPC differentiation in vitro, an impairment that can be partially reversed by rapamycin treatment, which modifies astrocyte senescence-associated signaling [139]. In addition to EV-mediated communication, astrocytes supply essential lipids and cholesterol for myelin synthesis, remodel the extracellular matrix, and form functional gap junctions with oligodendrocytes, collectively shaping the microenvironment that supports remyelination [140,141].

Although oligodendrocyte-derived EVs have been implicated in axonal metabolic support in neurodegenerative contexts, their contribution to post-stroke remyelination remains largely unexplored [142]. Given the close functional coupling between oligodendrocytes, axons, and astrocytes within WM tracts, future studies should determine whether oligodendrocyte-derived EVs participate in intercellular signaling networks that regulate myelin repair after ischemic injury.

Taken together, EVs released from microglia, endothelial cells, and astrocytes collectively influence oligodendrogenesis and remyelination following stroke, exerting either inhibitory or pro-regenerative effects depending on their cellular origin and cargo composition. By modulating OPC recruitment, survival, and differentiation while shaping the inflammatory and metabolic milieu, EV-mediated communication emerges as an important modulator of WM repair efficiency. These findings highlight EVs as promising therapeutic targets for enhancing remyelination and functional recovery after ischemic stroke, while also highlighting key knowledge gaps surrounding oligodendrocyte-derived EV signaling in cerebrovascular disease.

### 4.4. Axonal Growth and Synaptic Formation

Following ischemic stroke, the adult brain engages endogenous repair processes characterized by axonal sprouting, dendritic remodeling, and synaptic reorganization, particularly within peri-infarct regions and functionally connected circuits [143,144,145]. During the early post-stroke phase, a transient window of heightened plasticity, sharing features with critical developmental periods, permits structural rewiring, including increased dendritic spine turnover, axonal elongation, and synaptogenesis [146]. This plastic window supports reorganization of long-range motor pathways such as the corticospinal tract, where axons from the contralesional cortex sprout across the midline to re-innervate denervated spinal territories, a process associated with motor recovery in experimental models [147].

At the synaptic level, ischemic injury initially induces widespread synapse loss driven by glutamate excitotoxicity, mitochondrial dysfunction, and complement-dependent microglial pruning mediated by complement signaling (C1q/C3) [148,149]. Astrocytes regulate inhibitory synapse formation and remodeling through secretion of synaptogenic extracellular matrix components and modulation of synaptic phagocytosis. Astrocyte-derived extracellular matrix fragments such as the Neurocan C-terminal extracellular lectican fragment, promote inhibitory synapse formation, particularly on somatostatin-positive interneurons, thereby influencing excitatory–inhibitory balance [150]. Disruption of synaptic integrity contributes not only to motor deficits but also to post-stroke cognitive impairment, as altered synaptic plasticity impairs long-term potentiation and memory-related circuit function [151].

Neuroimmunological synapses, specialized interfaces between neurons and immune cells, further modulate synaptic remodeling after stroke. These interactions involve bidirectional signaling through cytokines, surface receptors, and vesicle-mediated communication, collectively influencing dendritic morphology, neuronal excitability, and circuit stability [152]. While immune cell–neuron interactions shape synaptic remodeling, EVs represent a parallel and potent mechanism of intercellular communication in the injured brain. Experimental evidence demonstrates that EVs are potent mediators of axonal and synaptic repair. Mesenchymal stromal cell-derived EVs enhance axonal elongation in cortical neurons via transfer of functional miRNAs that are incorporated into the recipient neuron’s Argonaute-2–containing RNA-induced silencing complex. Enrichment of these EVs with the miR-17–92 cluster further augments axonal growth by suppressing PTEN and activating mTOR/GSK-3β signaling, even in inhibitory post-injury environments [153]. Although MSCs are not NVU-resident cells, these studies provide mechanistic insight into how EV-borne miRNAs can directly drive axonal growth and synaptic remodeling following stroke.

Within the NVU, CEC-derived EVs contribute to axonal repair and synaptic preservation following ischemic injury. CEC-derived EVs are internalized by distal axons and undergo retrograde transport to neuronal soma, where transferred miRNAs (including miR-19a, miR-27a, and miR-298) accumulate and suppress intrinsic axonal growth inhibitors such as PTEN, RhoA, and semaphorin-6A, thereby promoting axonal elongation [44]. Genetic enrichment of CEC-derived EVs with miR-27a further augments axonal extension within peri-infarct cortex and the corticospinal tract, strengthening the pro-regenerative signaling capacity of endothelial EVs [154]. In parallel, endothelial EVs preserve synaptic structure by reducing inflammatory signaling and supporting expression of synaptic proteins such as PSD-95 and synaptophysin, consistent with protection against excessive synaptic loss [155].

Astrocyte-derived EVs modulate synaptic plasticity and neurite remodeling in both developmental and injury-associated contexts, with effects that depend strongly on astrocyte activation state [84,156]. Astrocyte-derived EVs promote neurite elongation, dendritic spine formation, and synapse assembly through delivery of regulatory proteins and RNAs that activate defined signaling cascades. For example, astrocyte EV-associated fibulin-2 drives synaptogenesis via TGF-β/Smad2 signaling [157], while additional studies implicate EV-mediated regulation of pathways such as Hippo signaling in structural neuronal remodeling [158]. Astroglia-derived EVs also carry synaptic and adhesion-associated proteins such as synapsin-I and HepaCAM, which promote neurite growth and dendritic organization via contact-dependent signaling mechanisms [159]. However, astrocyte EV cargo is highly stimulus dependent. Exposure of astrocytes to inflammatory signals such as IL-1β alters EV composition and impairs neuronal excitability and neurite outgrowth, underscoring the context-dependent nature of astrocyte-mediated synaptic remodeling [160].

EVs released from inflammatory microglia, enriched in TNF and miR-146a-5p, impair synaptic protein expression, reduce dendritic spine density, and amplify neuroinflammatory signaling by transferring regulatory cargo to neurons and suppressing synaptic targets such as synaptotagmin-1 and neuroligin-1 [161,162,163]. Microglia-derived EVs can also directly drive synaptic pruning by delivering complement component C1q to presynaptic sites, facilitating complement-tagged synapse elimination during post-injury remodeling [164]. In injury paradigms, presynaptic terminals may undergo vesicular fragmentation prior to microglial internalization, a process accompanied by complement tagging that further supports synaptic clearance [165]. In contrast, EVs derived from reparative or IL-4–stimulated microglia promote neuroprotective and plasticity-supportive signaling by transferring bioactive lipids and regulatory miRNAs associated with synaptic stabilization and structural repair [137,160]. Microglial regulation of synaptic remodeling is further shaped by C-X3-C motif chemokine receptor 1 (CX3CR1) and triggering receptor expressed on myeloid cells 2 (TREM2) activity, together with interleukin-10 (IL-10)–brain-derived neurotrophic factor (BDNF) signaling, integrating immune state with circuit-level repair [166].

Neuron-derived EVs also contribute to circuit reorganization by transmitting synaptogenic signals between interconnected neurons. BDNF stimulates the selective sorting of plasticity-associated miRNAs, including miR-132-5p and miR-218-5p, into neuron-derived small EVs, which are subsequently transferred to recipient neurons. These BDNF-induced EVs enhance excitatory synapse clustering, increase synaptic vesicle organization, and promote synchronous neuronal network activity in a miRNA-dependent manner, thereby propagating synaptogenic signaling beyond the site of initial BDNF stimulation [167]. Consistent with this, neuron-derived EVs contain synaptic and signaling molecules that modulate dendritic complexity and synapse maturation [18], supporting a role for EV-mediated communication in coordinating structural and functional plasticity across neuronal circuits.

Rather than acting in isolation, EVs derived from endothelial cells, astrocytes, microglia, neurons, and NSPCs form an integrated communication network that coordinates axonal growth and synaptic remodeling after stroke. By delivering cargo tailored to cellular activation state and injury context, including miRNAs, trophic regulators, lipids, and structural proteins, these vesicles modulate axon elongation, synapse formation, and pruning while limiting maladaptive plasticity. This EV-mediated coordination of neurovascular, immune, and neuronal responses positions EVs as important contributors to post-stroke circuit reorganization and potential targets for therapeutic strategies aimed at enhancing long-term functional recovery.

### 4.5. Anti-Inflammatory Effects in Chronic Neuroinflammation

Chronic neuroinflammation, persisting for weeks to months after ischemic stroke, represents a maladaptive extension of the acute immune response and is increasingly recognized as a major contributor to long-term neurological impairment. Experimental studies demonstrate sustained microglial activation and phagocytic activity within peri-infarct and necrotic regions well into the late chronic/recovery phase, indicating that inflammatory processes remain active long after infarct stabilization and tissue cavitation [168]. In parallel, chronic stroke is associated with prolonged blood–brain barrier dysfunction, persistent astrocytic reactivity, and continued production of pro-inflammatory mediators at both central and systemic levels [90,91]. These ongoing inflammatory signals are linked to impaired synaptic remodeling, WM degeneration, and progressive cognitive decline, suggesting that unresolved immune activation may constrain long-term structural and functional recovery.

A defining feature of chronic post-stroke inflammation is its self-perpetuating nature. Mitochondrial damage-associated molecular patterns (DAMPs), including cytochrome c and mitochondrial transcription factor A, are released from stressed or degenerating neurons and glia and activate pattern-recognition receptor signaling pathways, including Toll-like receptor and NF-κB cascades in microglia and astrocytes, thereby reinforcing feed-forward inflammatory loops [96]. Rather than supporting adaptive plasticity, this persistent low-grade inflammatory state disrupts synaptic homeostasis and neuroplasticity and is associated with progressive cognitive decline through mechanisms that overlap with, but are not identical to those described in neurodegenerative disorders [97]. These observations underscore the need for therapeutic strategies that selectively modulate chronic/recovery-phase inflammatory signaling rather than broadly suppressing early immune responses, which have shown limited clinical success.

Compared with angiogenesis, neurogenesis, and WM repair, the contribution of EVs to chronic post-stroke neuroinflammation remains less well defined, reflecting both biological complexity and a relative scarcity of longitudinal, stroke-specific EV studies. Nevertheless, emerging evidence indicates that NVU-derived EVs act as intercellular messengers capable of shaping glial activation states and inflammatory signaling pathways during the chronic/recovery phase. CEC-derived EVs have been identified as potential endogenous modulators of sustained neuroinflammation. In inflammatory models relevant to cerebrovascular injury, endothelial EVs delivering miR-672-5p suppress transforming growth factor beta-activated kinase 1 (TAK1)–NF-κB signaling through targeting TGF-β-activated kinase binding protein 2 (TAB2), promote polarization toward anti-inflammatory microglial phenotypes, and enhance autophagic degradation of NLRP3 inflammasome components [108]. Complementary studies further demonstrate that cerebral microvascular endothelial EVs preserve blood–brain barrier integrity and limit leukocyte infiltration under inflammatory conditions, processes directly relevant to chronic stroke pathology where ongoing barrier dysfunction perpetuates immune activation [105]. Together, these findings suggest that endothelial EVs may participate in restraining chronic inflammatory signaling rather than initiating immune responses.

Astrocyte-derived EVs display pronounced context dependence during chronic neuroinflammation. Under pro-inflammatory stimulation with TNFα or IL-1β, astrocytes modify the miRNA cargo of their EVs, enriching them with miR-125a-5p and miR-16-5p, which target neurotrophin receptor signaling pathways in recipient neurons [77,169]. Transfer of these EV-associated miRNAs suppresses NTRK3 (TrkC) and downstream Bcl2 expression, leading to reduced dendritic complexity, decreased neuronal firing rates, and dampened network activity [170]. These findings indicate that inflammatory astrocyte-derived EVs can actively remodel synaptic structure and excitability through post-transcriptional regulation of neurotrophic signaling. In contrast, astrocyte-derived EVs generated under ischemic preconditioning or reparative conditions carry protective cargo, including miR-92b-3p, which attenuates neuronal apoptosis and improves survival following oxygen-glucose deprivation (OGD) [50]. This duality underscores that astrocyte-derived EVs do not uniformly promote degeneration or repair but instead reflect the activation state of the parent astrocyte and the surrounding inflammatory milieu.

Microglia-derived EVs similarly function as phenotype-specific signaling units during chronic neuroinflammation. In response to inflammatory stimuli such as lipopolysaccharide, microglia release EV populations enriched in pro-inflammatory cytokines and immune-associated proteins, thereby propagating inflammatory signaling within the neural microenvironment [171]. Conversely, microglial EVs enriched in regulatory miRNAs such as miR-124 have been shown to suppress neuronal inflammation and promote neurite outgrowth following injury [172]. Together, these observations suggest that microglial EVs transmit immune-state information across the NVU, reinforcing either inflammatory persistence or facilitating resolution depending on the activation profile of the parent cell.

Taken together, EVs derived from endothelial cells, astrocytes, and microglia contribute to the regulation of chronic neuroinflammation after stroke by modulating glial phenotypes, inflammatory signaling pathways, autophagic mechanisms, and blood–brain barrier integrity. Rather than uniformly suppressing inflammation, EV-mediated communication appears to fine-tune immune responses in a context- and cell-state-dependent manner. Importantly, the limited availability of longitudinal, stroke-specific EV studies highlights a critical translational gap: defining how EV signaling evolves across chronic recovery and determining whether EVs actively drive immune resolution or primarily reflect ongoing inflammatory states. Addressing this gap will be essential for developing phase-specific immunomodulatory strategies that mitigate chronic neuroinflammatory injury without compromising adaptive repair processes. During the chronic/recovery phase, EV-mediated signaling does not operate within isolated vascular, glial, or neuronal compartments but instead reflects integrated NVU-level coordination in which angiogenesis, white matter repair, synaptic remodeling, and inflammatory resolution converge through shared regulatory pathways.

## 5. Conclusions, Challenges, and Future Directions

NVU-derived EVs participate in a dynamic and context-dependent intercellular signaling network that reflects the evolving cellular state of the post-stroke brain. Across the acute and chronic/recovery phases, EV cargo composition mirrors endothelial activation, glial reactivity, metabolic stress, and reparative signaling, positioning these vesicles as both reporters and modulators of stroke pathophysiology. Rather than functioning as uniform drivers of recovery, NVU-derived EVs influence the balance between injury propagation and repair through the transfer of context-dependent molecular signals that shape inflammation, vascular remodeling, neuronal survival, and circuit reorganization [173].

A central theme emerging from current evidence is the dual nature of EV signaling. In the early phase after stroke, NVU-derived EVs may contribute to thromboinflammation, endothelial dysfunction, and BBB disruption. In later phases, EV populations increasingly carry cargos associated with angiogenesis, neurogenesis, oligodendrogenesis, and synaptic plasticity. This temporal shift underscores the importance of clearly distinguishing EVs as pathogenic mediators in acute injury from EVs as potential therapeutic agents in later reparative contexts—a distinction that remains essential for both mechanistic interpretation and translational development [173].

Despite strong preclinical interest, several challenges currently limit clinical translation. Substantial methodological heterogeneity persists across EV isolation, purification, and characterization approaches. Even with increasing adherence to MISEV recommendations, variability in yield, purity, and reported cargo composition complicates cross-study comparisons and reproducibility [12]. Continued refinement of standardized reporting practices and transparent methodological disclosure are essential to ensure rigor, particularly as neural-derived EVs move toward therapeutic evaluation. In addition, the emphasis on miRNA cargo throughout this review reflects the current state of the literature, in which miRNA content of EVs has been more extensively characterized than protein or lipid cargo in stroke-relevant models. While EV lipids and proteins undoubtedly contribute to signaling, comparatively fewer mechanistic studies have defined their functional roles in ischemic stroke, resulting in a stronger evidence base for miRNA-mediated effects.

Effective EV delivery remains another major barrier. Systemic administration frequently results in rapid clearance and preferential accumulation in peripheral organs such as liver and spleen, reducing brain-specific targeting efficiency [174,175]. Intranasal and intrathecal approaches show promise but require further optimization. Engineering strategies aimed at enhancing surface targeting ligands or modifying EV uptake mechanisms may improve brain delivery, yet these approaches introduce additional complexity in scalability, batch consistency, and regulatory oversight [174]. Beyond surface modification, cargo engineering strategies such as selective miRNA enrichment or genetic manipulation of parent cells to alter EV content are increasingly explored to enhance therapeutic specificity [176].

Biological variables further complicate interpretation and translation. Sex-specific differences in immune responses and epigenetic regulation may influence stroke outcomes and EV-mediated signaling dynamics, yet these variables remain underexplored in EV-focused studies [177]. Similarly, aging is associated with altered post-transcriptional regulation and proteostatic shifts that may modify EV cargo composition and recipient-cell responsiveness [178]. Aging is also associated with delayed resolution of post-stroke inflammation, altered microglial polarization dynamics, and impaired regenerative signaling, which may shift optimal EV dosing windows, therapeutic responsiveness, and duration of treatment required in aged models [177]. Greater attention to age- and sex-specific responses in preclinical models will be essential for generating clinically relevant therapeutic frameworks.

Beyond technical and biological hurdles, EV-based interventions require careful consideration of potential risks. Because endogenous NVU-derived EVs participate in inflammatory and thrombotic signaling pathways following stroke [173], therapeutic manipulation must account for the possibility of unintended immune activation, pro-thrombotic signaling, or maladaptive remodeling. A balanced translational strategy therefore demands rigorous functional validation, dose optimization, and longitudinal safety assessment.

Looking forward, progress in this field will depend less on expanding descriptive catalogs of EV cargo and more on developing integrative frameworks that define how EVs function as temporal state reporters and bidirectional injury–repair mediators within the NVU (Table 4). Standardized temporal schemas, explicit species annotation, careful distinction between stroke-specific and extrapolated CNS evidence, and adherence to evolving MISEV guidelines [12] will be critical to maintaining interpretive rigor.

NVU-derived EVs represent an emerging component of stroke pathophysiology and recovery. However, their clinical utility will ultimately depend on methodological standardization, phase-specific therapeutic design, transparent reporting, and cautious translation grounded in reproducible and stroke-specific evidence.

## Figures and Tables

**Table 1:** NVU-Derived Extracellular Vesicles in Acute Ischemic Stroke. Grouped summary of EV signaling from major NVU cell types during the acute phase of ischemic stroke (hours to ~3 days post-ischemia). Evidence derives mainly from MCAO/tMCAO and OGD models, with human data noted where applicable. Listed cargo represents representative regulatory RNAs and pathways. Functional categories reflect aggregated roles of EV signaling. Effects represent convergent mechanisms across models and do not imply uniform causality across species or stroke contexts. Unless explicitly noted, evidence derives from preclinical or in vitro systems and should not be interpreted as demonstrated clinical efficacy in human stroke.

EV Source	Model/Context	Representative Cargo/Pathway	Functional Effect/Mechanism	Citations
CEC-EVs	OGD, MCAO, tMCAO/R, BBB models	miR-1290; PDGF-B/PDGFRβ; Ang1/Ang2-Tie2 signaling	Protect neurons and stabilize BBB during early vascular stress	[39,40,41]
Astrocyte-derived EVs (protective phenotype)	MCAO, I/R, OGD neuronal models	miR-378a-5p, miR-34c, miR-190b, miR-628	Suppress neuronal death pathways (pyroptosis, apoptosis, autophagy) and promote recovery signaling	[42,43,44,45]
Astrocyte-derived EVs (barrier & inflammatory modulation)	OGD endothelial stress; inflammatory stimulation	miR-27a-3p; miR-125a-5p; miR-16-5p	Stabilize endothelial barrier and modulate neuronal activity during inflammation	[46,47]
Neuron-derived EVs	tMCAO	miR-98	Prevent phagocytosis of stressed but viable neurons	[48]
Microglia-derived EVs (M1)	MCAO/R	circSTRN3 → NF-κB signaling	Amplify neuroinflammation and astrocyte neurotoxicity	[49]
Microglia-derived EVs (M2)	tMCAO	miR-135a-5p to TXNIP/NLRP3 inhibition	Suppress inflammasome activation and neuronal injury	[50]
Pericyte-associated EVs	Human acute stroke plasma	Pericyte-marker vesicles	Reflect acute vascular signaling changes (correlative evidence)	[51,52]
Thrombus-associated endothelial EVs	Human thrombectomy clots	Procoagulant phosphatidylserine & tissue factor	Promote microvascular injury and secondary occlusion	[53]

**Table 2:** Phase-Associated Functional Tendencies of NVU-Derived Extracellular Vesicles after Ischemic Stroke. Classification of predominant NVU-derived EV sources and representative cargo across acute, subacute, and chronic phases of ischemic stroke (timeframes primarily based on rodent ischemia models). Listed cargo includes miRNAs (e.g., miR-126, miR-124, miR-23a-5p, miR-132), inflammatory mediators (TNF-α, IL-6), growth factors (VEGF, FGF-2, PDGF-BB), complement proteins, and pathway regulators (PI3K/Akt, PTEN/RhoA, NF-κB). Functional tendencies reflect model-specific experimental findings and are state-dependent rather than temporally exclusive.

Stroke Phase	Predominant EV Sources (State-Dependent)	Dominant Functional Tendencies	Translational Considerations	Representative Cargo/Pathways
Acute (hours–≤7 d, rodent models)	Pro-inflammatory microglia (M1-like), reactive astrocytes, injured neurons, circulating/clot-derived EVs	Amplify inflammation; promote BBB permeability; propagate neuronal stress; pro-thrombotic signaling	Context-dependent; early intervention may require suppression of pathogenic EV signaling	TNF-α; IL-6; circSTRN3 → MAVS/NF-κB; miR-146a; miR-125a-5p; MMP-9; tissue factor
Subacute (~3–14 d, model-dependent)	Endothelial cells (CECs/EPCs), pericytes, transitioning glia	Angiogenesis; vascular remodeling; glial phenotype transition; early WM support	Window for modulating vascular–glial coupling and inflammatory resolution	miR-126 → PI3K/Akt; VEGF; PDGF-BB; Ang1/2; miR-27a; CRYAB; miR-92b-3p
Chronic (weeks–months, species-dependent)	Reparative microglia (M2-like), NSCs/NPCs, astrocytes, endothelial cells	Neurogenesis; oligodendrogenesis; synaptic remodeling; sustained immunomodulation	Candidate window for regenerative and biomimetic EV-based strategies; requires dosing and targeting optimization	miR-124; miR-23a-5p; miR-132; miR-218; FGF-2; Apo-D; HSP70; PTEN/RhoA modulation

**Table 3:** NVU-Derived Extracellular Vesicles in Chronic Stroke. Grouped summary of EV signaling from major NVU cell types during the chronic phase of ischemic stroke (weeks to months post-injury). Evidence derives primarily from MCAO/tMCAO and OGD models, with complementary niche and vascular-risk studies included where they directly inform late-stage repair mechanisms. Listed cargo represents representative regulatory RNAs and signaling pathways. Functional categories reflect aggregated roles of EV signaling. Effects represent convergent mechanisms across experimental models and do not imply uniform causality across species, injury severity, or glial activation states. Unless explicitly noted, evidence derives from preclinical or in vitro systems and should not be interpreted as demonstrated clinical efficacy in human stroke.

EV Source	Model/Context	Representative Cargo/Pathway	Functional Effect/Mechanism	Citations
CEC-derived EVs	Experimental stroke, vascular OGD, aging vascular risk models	Pro-angiogenic miRNAs; PTEN/RhoA targeting; MYD88/TSP1 suppression; BBB-stabilizing signaling	Promote reperfusion, restore BBB integrity, regulate neurogenesis niches, and coordinate vascular–neuronal remodeling	[86,87,88,89,90,91]
Pericyte-derived EVs	Hypoxic vascular stress and conditioning	Pro-angiogenic regulatory factors	Support endothelial adaptation and microvascular stabilization	[92,93]
Astrocyte-derived EVs	Ischemia–reperfusion and repair phase; Cytokine activation, aging	Trophic remodeling signals; FGF-2, VEGF, Apo-D; miR-92b-3p; miR-125a-5p; miR-16-5p; altered trophic cargo	Promote circuit reorganization, oligodendrocyte maturation, and neuronal survival; Impair dendritic complexity and OPC differentiation	[47,71,93,94,95,96,97]
Microglia-derived EVs (M1-like)	Post-stroke reparative microglia	TGF-β/Smad2/3; miR-23a-5p; miR-124; TNF → TNFR2; endocannabinoids	Promote neurite growth, WM repair, anti-inflammatory polarization, and tissue remodeling	[98,99,100,101,102,103,104,105]
Microglia-derived EVs (M2-like)	Injury and chronic inflammatory conditions	Cytokine-rich inflammatory EV cargo	Impair remyelination and sustain neuroinflammation	[106,107,108]
NPC-derived EVs	MCAO and stem-cell niche models	Anti-apoptotic and paracrine proteomic signaling	Expand NPC pool and promote neurogenesis-associated recovery	[89,109,110]

**Table 4:** Translational Priorities for NVU-Derived EV-Based Strategies in Ischemic Stroke. Overview of key translational focus areas for advancing NVU-derived EVs toward clinical application. Priorities reflect challenges discussed in this review, including targeting specificity, cargo standardization, delivery optimization, temporal stratification by stroke phase, incorporation of biological variables (age, sex, inflammatory state), and adherence to MISEV-compliant isolation and GMP-grade production standards. Listed strategies represent preclinical and early translational considerations rather than established clinical interventions.

Priority Area	Refined Focus (Aligned with Current Evidence)
EV Engineering	Surface modification to enhance brain/NVU cell targeting (e.g., endothelial, OPC, neuronal uptake); optimization of biodistribution and cellular specificity
Cargo Optimization	Controlled enrichment of defined regulatory cargo (miRNAs, proteins, lipids); evaluation of gene-editing or RNA-loading approaches with safety validation
Delivery Strategies	Optimization of intranasal, intrathecal, and biomaterial-assisted delivery (e.g., hydrogel-based retention); minimizing systemic sequestration
Diagnostic Integration	Phase-specific EV profiling as biomarkers of thromboinflammation, vascular remodeling, or chronic neuroinflammation
Clinical Trial Design	Temporal stratification by stroke phase; incorporation of age, sex, and inflammatory status; longitudinal safety monitoring
Standardization & Regulation	MISEV-compliant isolation and reporting; GMP-grade scalable production; batch consistency; transparent cargo characterization

## Data Availability

No new data were created or analyzed in this study. Data sharing is not applicable to this article.

## Abbreviations

The following abbreviations are used in this manuscript:

Ang-1	angiopoietin-1
Ang-2	angiopoietin-2
ApoD	apolipoprotein D
ARHGAP25	rho GTPase-activating protein 25
AQP4	aquaporin-4
BBB	blood–brain barrier
BDNF	brain-derived neurotrophic factor
C1q	complement component 1q
Cav-1	caveolin-1
CECs	cerebral endothelial cells
CNS	central nervous system
COX-2	cyclooxygenase-2
DAMPs	damage-associated molecular patterns
DCX	doublecortin
EPCs	endothelial progenitor cells
ERK	extracellular signal-regulated kinase
EVs	extracellular vesicles
EVT	endovascular thrombectomy
FGF-2	fibroblast growth factor-2
GFAP	glial fibrillary acidic protein
Hsc70	heat shock cognate protein 70
IgG	immunoglobulin G
IL-1β	interleukin-1 beta
IL-1RA	interleukin-1 receptor antagonist
IL-4	interleukin-4
IL-6	interleukin-6
IL-10	interleukin-10
Iba1	ionized calcium-binding adaptor molecule
iNPCs	induced neural progenitor cells
JAK1	Janus kinase 1
MAVS	mitochondrial antiviral signaling protein
L1CAM	L1 adhesion molecule
MCP-1	monocyte chemoattractant protein-1
MISEV	minimal information for studies of extracellular vesicles
miRNAs	microRNAs
MMP-9	matrix metalloproteinase-9
MSCs	mesenchymal stem cells
mTOR	mechanistic target of rapamycin
MVB	multivesicular body
NETs	neutrophil extracellular traps
NF-κB	nuclear factor kappa B
NLRP3	NOD-like receptor family pyrin domain-containing 3
NPCs	neural progenitor cells
NSCs	neural stem cells
NSPCs	neural stem/progenitor cells
NVU	neurovascular unit
OGD	oxygen-glucose deprivation
OPCs	oligodendrocyte progenitor cells
PAFR	platelet-activating factor receptor
PDGF	platelet-derived growth factor
PDGFRβ	platelet-derived growth factor receptor beta
PECAM-1	platelet endothelial cell adhesion molecule-1
PI3K	phosphoinositide 3-kinase
PSD-95	postsynaptic density protein 95
PTEN	phosphatase and tensin homolog
RhoA	Ras homolog family member A
Sema6A	semaphorin 6A
SGZ	subgranular zone
Smad2/3	SMAD family member 2/3
Sox2	SRY-box transcription factor 2
STAT3	signal transducer and activator of transcription 3
SVZ	subventricular zone
TAB2	TGF-β-activated kinase binding protein 2
TAK1	transforming growth factor beta-activated kinase 1
Tie2/TEK	tyrosine kinase with immunoglobulin-like and EGF-like domains 2
TLR7	toll-like receptor-7
tMCAO	transient middle cerebral artery occlusion
TNF	tumor necrosis factor
TNF-α	tumor necrosis factor alpha
TNFR2	tumor necrosis factor receptor 2
tPA	tissue plasminogen activator
TREM2	triggering receptor expressed on myeloid cells 2
VEGF	vascular endothelial growth factor
vWF	von Willebrand factor
WM	white matter
ZO-1	zonula occludens-1
